# Establishment and Validation of a New Predictive Model for Insulin Resistance based on 2 Chinese Cohorts: A Cross-Sectional Study

**DOI:** 10.1155/2022/8968793

**Published:** 2022-10-17

**Authors:** Shi Zhang, Xin-Cheng Wang, Jing Li, Xiao-He Wang, Yi Wang, Yan-Ju Zhang, Mei-Yang Du, Min-Ying Zhang, Jing-Na Lin, Chun-Jun Li

**Affiliations:** ^1^Department of Endocrinology, Health Management Center, Tianjin Union Medical Center, Nankai University Affiliated Hospital, Tianjin, China; ^2^School of Medicine, Nankai University, Tianjin, China; ^3^Tianjin Centers for Disease Control and Prevention, Tianjin, China

## Abstract

**Background:**

Visceral adiposity plays a key role in the development of insulin resistance (IR), so surrogate index that can indicate visceral obesity may have higher predictive value for IR. This study aimed to establish and validate a new predictive model including indicator of visceral obesity for IR.

**Methods:**

The study population consisted of two cohorts. The derivation cohort was a group of 667 patients with newly diagnosed type 2 diabetes and the population undergoing a routine health checkup was the validation cohort. The predictive model was established by the logistic regression analysis. Its value for predicting IR was compared with other surrogate indices by the receiver operating characteristic curve.

**Results:**

The odds ratio (OR) of age, visceral fat area (VFA), triglyceride (TG), fasting plasma glucose (FPG), and alanine aminotransferase (ALT) for IR was 1.028 (95% CI, 1.008–1.048) (*P* < 0.01), 1.016 (95% CI, 1.009–1.023) (*P* < 0.001), 1.184 (95% CI, 1.005–1.396) (*P* < 0.05), 1.334 (95% CI, 1.225–1.451) (*P* < 0.001), and 1.021 (95% CI, 1.001–1.040) (*P* < 0.05). The formula of the predictive model was (0.0293 × age + 1.4892 × Ln VFA + 0.4966 × Ln TG + 2.784 × Ln FPG + 0.6906 × Ln ALT)/2. The area under the curve was the largest among all the previously reported predictors.

**Conclusions:**

This study established and validated a predicting model for IR and confirmed its predictive value in comparison with other surrogate indicators, which will offer a simple and effective tool to measure IR in future large population studies.

## 1. Introduction

The prevalence of diabetes is increasing annually worldwide. There are around 463 million diabetic patients globally, the vast majority of whom are type 2 diabetes mellitus (T2DM) [1]. In China, the rise of diabetes prevalence is even more dramatic, which rose from less than 1% in 1980 to 12.8% in 2018 [2]. Obesity contributes to insulin resistance (IR) via a series of biological pathways, including increasing the secretion of proinflammatory cytokines, reducing adiponectin production, and enhancing the metabolically deteriorating effects of exosomes, which will ultimately lead to the development of T2DM [3].

Hyperinsulinemic euglycemic clamp technique (HEC) was first proposed by DeFronzo in 1979 and is regarded as the gold standard for assessing IR currently [4]. However, there exist some factors such as the complexity, high cost, and consumption of time that limit its wide application in large epidemiological studies. Homeostatic model assessment of insulin resistance (HOMA-IR) calculated by the concentration of plasma insulin and glucose is considered to be a reliable predictor for IR; whereas insulin is not a routine measurement in clinical practice and its determination has not been standardized [5]. Under such circumstances, a number of simple predictors for IR such as the triglyceride (TG) and glucose (TyG) index, visceral adiposity index (VAI), the TG to high-density lipoprotein cholesterol (HDL-C) ratio (THR), and lipid accumulation product (LAP) have been put forward [6]. However, the predictive value of these surrogate indicators may vary across different ethnic groups and cohorts with different metabolic disorders [7].

Therefore, this study aimed to establish a new predictive model for IR in a Chinese cohort with newly diagnosed T2DM, validate the model in another cohort undergoing annual routine health checkup and further explore the predictive value of the model compared with the predictors reported in previous studies.

## 2. Methods

### 2.1. Study Population

The study population consisted of two cohorts. The derivation cohort came from a group of 921 patients with newly diagnosed T2DM admitted to the Department of Endocrinology of Tianjin Union Medical Center during May 2017 to December 2020. The diagnosis of T2DM was based on the 2020 Chinese Guidelines for the Prevention and Treatment of T2DM [8]. The patients were excluded if they were complicated by serious cerebrocardiovascular disease, unstable mental state and refused to provide the informed consent. Ultimately, 667 patients with new-onset T2DM constituted the derivation cohort. The group, including 1050 individuals who underwent a routine health checkup in the Health Management Center of Tianjin Union Medical Center in December 2021, was the target population for the validation cohort. At last, a total of 271 people were enrolled in the validation cohort after 779 were excluded for lack of insulin measurement, incomplete information and unwillingness to provide the written informed consent.

**Figure 1:**
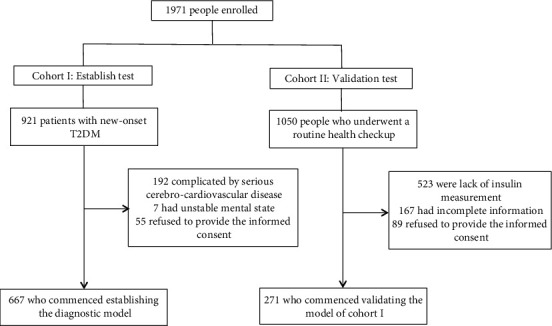
Flowchart of the study design and participants recruitment in this study.

The flowchart outlining the study design and sample collection was shown in Figure 1. The Medical Ethics Committee of Tianjin Union Medical Center approved the study (approval number: 2021C06) and all subjects agreed to participate in the study and signed the informed consent.

### 2.2. Sociodemographic and Clinical Information Collection

Detailed information on sociodemographic characteristics including age, gender, smoking, and drinking status were collected. According to the World Health Organization (WHO), smoking was defined as ≥1 cigarette per day for more than 6 months and drinking was defined as ≥3 times per week for more than 6 months [9]. Other information on blood pressure and past medical history such as hyperuricemia, dyslipidemia, nonalcoholic fatty liver disease (NAFLD), and hypertension were extracted from the medical records.

### 2.3. Anthropometric and Body Composition Measurement

All participants underwent the anthropometrical assessments including height, weight, and waist circumference (WC) in fasting state with light clothes and barefoot. The calculation of body mass index (BMI) was based on the value of height in meters and weight in kilograms. WC was measured at the midpoint between the iliac crest and the costal margin as the WHO recommended [10]. The direct segmental multifrequency bioelectrical impedance analysis (BIA) method (InBody 770, Bio-space Inc., Korea), which has been considered to be a reliable and noninvasive method for assessment of body composition currently, was adopted to evaluate the visceral fat area (VFA). Detailed procedures for body composition measurement had been described in our previous study [11].

### 2.4. Biochemical Assessment

Blood samples were taken after an overnight fasting and before hypoglycemic treatments were given. Fasting plasma glucose (FPG), TG, low density lipoprotein cholesterol (LDL-C), HDL-C, uric acid (UA), alanine aminotransferase (ALT), and aspartate aminotransferase (AST) were measured by an automatic biochemical analyzer (TBA-120FR, Toshiba, Tokyo). Fasting insulin (FINS) was determined by chemiluminescent immunoassay.

### 2.5. Definition of IR and Surrogate Indices

IR which was calculated as FINS (IU/L) × FPG (mmol/L)/22.5 was defined by HOMA-IR > 2.69 [12].

The calculation formulas for TyG index, LAP, VAI, and THR were as follows: TyG index = Ln (TG (mmol/L) × FBG (mmol/L)/2) [13]; LAP = (WC (cm) − 65) × (TG (mmol/L)) (in men), (WC (cm) − 58) × (TG (mmol/L)) (in women) [14]; VAI = (WC (cm)/39.68 + 1.88 × BMI (kg/m^2^)) × (TG (mmol/L)/1.03) × (1.31/HDL-C (mmol/L)) (in men), (WC (cm)/36.58 + 1.89 × BMI (kg/m^2^)) × (TG (mmol/L)/0.81) × (1.52/HDL-C (mmol/L)) (in women) [15]; THR = TG (mmol/L)/HDL (mmol/L) [16].

### 2.6. Statistical Analysis

Statistical analyses were performed using SPSS statistical software (version 26.0, IBM Corp.). Continuous data were expressed as mean ± standard deviation or median (interquartile range). Categorical variables were presented as numbers or percentages. Continuous data were analyzed using independent two-sample *t*-test or unpaired Mann–Whitney *U* test. The chi-squared test was used for analysis of categorical data. The relationships between various factors and IR were analyzed by logistic regression analysis, which were presented as odds ratio (OR) and 95% confidence interval (CI). With regard to multicollinearity, a representative one was selected from highly correlated variables. The Hosmer–Lemeshow test was adopted to evaluate whether the regression model fit the data correctly. The receiver operating characteristic (ROC) curve was used to evaluate the predictive value of the model for IR. *P* < 0.05 was considered statistically significant.

## 3. Results

### 3.1. Clinical Characteristics of the Two Study Cohorts

The whole study population included two cohorts, each of which was divided into the IR group and non-insulin resistance (non-IR) group based on the presence of IR or not. The clinical characteristics of the two cohorts are demonstrated in Table 1. We could see that BMI, VFA, and WC were significantly higher in the IR group than the non-IR group in both cohorts (*P* < 0.001). No matter which cohort they were in, subjects in the IR group had much higher parameters indicating unfavorable cardiometabolic outcome such as FPG, TG, ALT, and AST compared with those in the non-IR group (*P* < 0.05). The FINS level was higher in the IR group than the non-IR group (*P* < 0.001).

In addition, cardiometabolic disorders including hyperuricemia, dyslipidemia, NAFLD, and hypertension were more common in the IR group. Surrogate indicators for IR such as TyG index, LAP, and VAI were obviously higher in the IR group than the non-IR group in both the two cohorts (*P* < 0.001), except that THR did not reach statistical significance in the derivation group.

### 3.2. Univariate Logistic Regression Analysis in the Derivation Cohort

The univariate logistic regression analysis was performed in the derivation cohort. Results suggested that BMI, VFA, WC, FPG, FINS, TG, ALT, AST, UA, and the history of NAFLD were significantly associated with IR. Their ORs were 1.177 (95% CI, 1.116–1.241), 1.018 (95% CI, 1.012–1.025), 1.052 (95% CI, 1.034–1.070), 1.312 (95% CI, 1.210–1.422), 1.907 (95% CI, 1.701–2.138), 1.365 (95% CI, 1.156–1.613), 1.019 (95% CI, 1.009–1.030), 1.026 (95% CI, 1.007–1.046), 1.003 (95% CI, 1.000–1.005), and 1.964 (95% CI, 1.379–2.796), respectively. All *P* values were less than 0.05. The results are shown in Table 2.

### 3.3. Multivariate Logistic Regression Analysis and the Establishment of the New Predictive Model

Variables with statistical significance in univariate logistic regression analysis were further analyzed by multivariate logistic regression analysis. Multicollinearity analysis of the independent variables demonstrated that there were collinearity relations between BMI, WC, and other variables, the variance inflation factor (VIF) of which were 5.757 and 9.749. Thus, the above two variables were removed. Meanwhile, the age that was generally recognized to have an effect on IR but was not statistically significant in the univariate analysis was included in the establishment of the model.

Results of multivariate logistic regression analysis showed the ORs of age, VFA, TG, FPG, and ALT for IR were 1.028 (95% CI, 1.008–1.048) (*P* < 0.01), 1.016 (95% CI, 1.009–1.023) (*P* < 0.001), 1.184 (95% CI, 1.005–1.396) (*P* < 0.05), 1.334 (95% CI, 1.225–1.451) (*P* < 0.001), and 1.021 (95% CI, 1.001–1.040) (*P* < 0.05). Finally, the formula of the new predictive model was (0.0293 × age + 1.4892 × Ln VFA + 0.4966 × Ln TG + 2.784 × Ln FPG + 0.6906 × Ln ALT)/2 [17]. The results are presented in Table 3.

### 3.4. Evaluation and Validation of the Predictive Model

**Figure 2:**
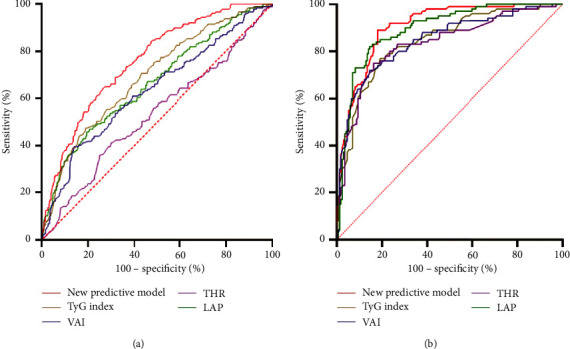
ROC curve of each index for predicting IR. (a) ROC curve for predicting IR in patients with T2DM. (b) ROC curve for predicting IR in healthy subjects. ROC: receiver operating characteristic, IR: insulin resistance, T2DM: type 2 diabetes mellitus, triglyceride to high-density lipoprotein cholesterol ratio, and LAP: lipid accumulation product.

The efficacy of the new model for prediction of IR was compared with the surrogate indices reported in the previous studies by ROC analysis. The area under the curve (AUC) of the predictive model in the derivation cohort was 0.77, superior to that of TyG index, LAP, VAI, and THR, which was 0.69, 0.66, 0.63, and 0.53, respectively. In the validation cohort, the AUC of the new model, TyG index, LAP, VAI, and THR was 0.91, 0.84, 0.89, 0.85, and 0.83, which suggested that the new predictive model still had the largest AUC among all the surrogate markers. Moreover, the specificity of the new model in the derivation and validation cohort was 0.81 and 0.89, which was the highest among all the predictors. The cut-off values of the new model were 8.18 in the derivation cohort and 7.46 in the validation cohort. The results are displayed in Table 4 and Figure 2.

## 4. Discussion

IR is crucial in the development of T2DM and other obesity-related diseases. HEC and HOMA-IR, which are considered to be the most accurate methods for IR evaluation, have not been widely used in clinical practice until now due to their complicated procedure, high price, and unstandardized measurement. To find an indicator with high predictive value and simplicity for the assessment of IR has been the focus of research in the past few decades. TyG index calculated by the levels of TG and FPG was first proposed by Simental–Mendia et al. in 2008 and verified by subsequent studies that it was a reliable and simple proxy for predicting IR [13, 18, 19]. The data from the Third National Health and Nutrition Examination Survey conducted in the United States demonstrated that LAP, which combined abdominal adiposity and circulating TG concentration, was much superior for predicting diabetes than BMI [14]. Recently, several studies found LAP could be used as an effective predictor of IR and NAFLD, and what's more, it had a relatively higher predictive value than traditionally applied surrogate indices [6, 18, 20]. VAI deriving from a formula based on anthropometric and biochemical parameters could indirectly express visceral adipose function and was a useful marker for evaluating IR in clinical practice [15, 21]. THR, the ratio of TG to HDL-C, has also been proved by some studies that it is a simple marker to identify IR and predict T2DM [16, 22]. In this study, we assessed the abilities of TyG index, LAP, VAI, and THR for predicting IR and found they were effective predictors of IR, as previous studies had suggested. However, the new predictive model mainly using VFA and biochemical indices based on a cohort with new-onset T2DM in our study showed the strongest predictive power among all the reported surrogate indices. In addition, the model was further validated in another cohort undergoing an annual health checkup and it still demonstrated the highest predictive efficacy.

The new predictive model demonstrated better ability to predict IR compared with all the other previously reported indicators in this study, the underlying reason of which might be that we combined VFA and circulating biochemical parameters. As it is well known, visceral adiposity plays a key role in the development of IR. Thus, surrogate markers that take into account visceral adiposity may show a higher predictive value than those only using some biochemical indices. The study conducted by Huang et al. had confirmed that LAP calculated by WC and TG had the largest AUC and highest specificity for prediction of IR in a middle-aged Chinese population [6], which might be due to that WC could reflect abdominal obesity to some extent; whereas WC is not a precise indicator of visceral fat and VFA can accurately indicate visceral fat deposition. Therefore, the predictive ability of the new model in this study surpassed that of LAP and was the most robust among all the surrogate indicators.

In this study, the clinical characteristics of the two study cohorts were analyzed firstly. Results suggested that IR was closely associated with obesity and cardiometabolic abnormalities, which was consistent with previous studies had shown [23, 24]. The new predictive model was established by logistic regression analysis. Risk factors of IR were first screened out by univariate logistic regression analysis, and then multivariate logistic regression analysis was performed. Four variables including VFA, TG, FPG, and ALT, which were associated with IR demonstrated by previous studies, were consistently identified as risk factors of IR in our study. VFA reflecting visceral adiposity has been confirmed to be strongly related to IR and the potential mechanism of this correlation is that visceral fat accumulation will increase the release of inflammatory cytokines, cause a low-grade chronic inflammation, and finally lead to IR [25]. Diacylglycerol (DAG), an intermediate lipid in the synthesis of TG, can activate protein kinase C *ε*, inhibit insulin receptor signaling pathway, and cause the development of IR [26]. In human studies, it was also found that intrahepatic DAG and TG were strongly associated with IR and metabolic disorders [27]. IR is characterized by the weakening of the biological effect of insulin on lowering blood glucose, therefore elevated FPG indicates that IR may have occurred. Moreover, a study conducted by Matsumoto et al. suggested that decreased early-phase insulin secretion might be the initial abnormality in the progress from normal to impaired glucose tolerance and hyperglycemia might have resulted in IR in Japanese ethnics [28]. Thus, hyperglycemia and IR are closely linked with each other. The insulin resistance atherosclerosis study conducted in America found that ALT was independently correlated to IR after adjustment for confounding factors [29]. Elevated ALT is due to the accumulation of free fatty acids in hepatocytes in the presence of obesity and IR, suggesting nonalcoholic steatohepatitis may have occurred [30]. Notably, age was not statistically significant in the univariate logistic regression analysis; whereas a considerable body of evidence had confirmed that age was associated with the increased risk of IR and T2DM [31, 32], based on which age was included in the model. Finally, the predicting model composed of these five variables presented high predictive value in both the derivation and validation cohorts, the AUC and specificity of which ranked first among all the previously reported predictors.

This study proposed a new predictive model based on easily acquired and inexpensive parameters for IR measurement, which in the meanwhile showed high predictive ability. It will provide a new and powerful tool for evaluating IR in future epidemiologic and large-scale clinical studies. There are several limitations in this study. First of all, HOMA-IR instead of HEC was used to assess IR in this study and so it could not provide the association of the new model and the gold standard. Secondly, this study was conducted based on two Chinese cohorts of newly diagnosed T2DM and natural population undergoing annual health checkup. The efficacy of the model in population with other metabolic abnormalities and in other ethnic groups is unknown. Thirdly, the sample size in this study is relatively limited and it is a retrospective cross-sectional study, which cannot explain the causal relationship.

In conclusion, this study established and validated a new predicting model for IR in two different Chinese cohorts and further confirmed its predictive value in comparison with other surrogate indicators, which will offer a simple and effective way to measure IR in later large population studies. Prospective cohort studies with larger sample sizes in other ethnic population and other groups with different metabolic disorders are needed to further validate and verify our results.

## Figures and Tables

**Table 1:** Clinical characteristics of the two study cohorts.

Characteristics	Derivation cohort		*P* ^b^	Validation cohort		*P* ^b^
	IR (*N* = 490)	Non-IR (*N* = 177)		IR (*N* = 172)	Non-IR (*N* = 99)	
Male/female	271/219	103/74	0.507	66/106	35/64	0.621
Age (y)	58.17 ± 10.89	58.44 ± 10.87	0.783	33.52 ± 12.05	32.33 ± 7.48	0.377
Smoking, *n* (%)	206 (42.04)	86 (48.59)	0.132	63 (36.63)	44 (44.45)	0.205
Drinking, *n* (%)	150 (30.61)	60 (33.90)	0.420	51 (29.65)	32 (32.32)	0.646
Hyperuricemia, *n* (%)	48 (9.80)	11 (6.21)	0.150	49 (28.49)	6 (6.06)	<0.001
Dyslipidemia, *n* (%)	286 (58.37)	89 (50.28)	0.063	50 (29.07)	13 (13.13)	0.003
NAFLD, *n* (%)	261 (53.27)	65 (36.72)	<0.001	88 (51.16)	20 (20.20)	<0.001
Hypertension, *n* (%)	85 (17.35)	33 (18.64)	0.698	51 (29.65)	5 (5.05)	<0.001
BMI (kg/m2)	26.93 ± 3.79	24.85 ± 3.46	<0.001	33.34 ± 6.61	22.70 ± 4.07	<0.001
VFA (cm2)	107.00 ± 30.68	91.27 ± 28.72	<0.001	182.60 ± 58.22	81.74 ± 39.25	<0.001
WC (cm)	95.68 ± 11.69	89.67 ± 10.33	<0.001	109.69 ± 16.40	83.83 ± 11.16	<0.001
FPG (mmol/L)	9.28 ± 2.73	7.63 ± 2.42	<0.001	5.57 ± 1.58	4.63 ± 0.46	<0.001
FINS (*μ*U/mL)	13.50 (9.92–20.48)	5.89 (4.49–7.60)	<0.001	20.80 (15.74–29.75)	7.63 (6.42–10.04)	<0.001
TG (mmol/L)	1.80 (1.28–2.62)	1.37 (1.00–2.12)	<0.001	1.69 (1.14–2.10)	0.80 (0.66–1.06)	<0.001
HDL-C (mmol/L)	1.22 ± 0.34	1.20 ± 0.30	0.571	1.32 ± 0.29	1.64 ± 0.37	<0.001
LDL-C (mmol/L)	3.22 ± 0.83	3.12 ± 0.78	0.133	2.98 ± 0.66	2.70 ± 0.80	0.002
ALT (IU/L)	21.85 (14.90–35.55)	17.50 (13.25–25.10)	<0.001	31.05 (19.10–45.10)	16.00 (12.00–24.50)	<0.001
AST (IU/L)	16.60 (13.40–23.00)	15.60 (12.80–19.40)	0.005	24.40 (17.90–28.95)	18.00 (15.00–21.70)	<0.001
UA (*μ*mol/L)	302.82 ± 84.14	285.53 ± 83.26	0.019	381.62 ± 102.79	289.80 ± 85.01	<0.001
TyG index	2.15 ± 0.69	1.69 ± 0.64	<0.001	1.09 ± 0.32	0.70 ± 0.24	<0.001
LAP	61.83 (36.82–97.28)	38.49 (21.61–67.94)	<0.001	83.63 (54.09–113.47)	17.02 (11.61–32.90)	<0.001
VAI	2.74 (1.72–3.93)	1.91 (1.27–3.33)	<0.001	2.20 (1.44–3.21)	0.83 (0.62–1.30)	<0.001
THR	1.58 (1.05–2.34)	1.39 (0.96–2.43)	0.299	1.29 (0.87–1.73)	0.50 (0.38–0.73)	<0.001

(a) Continuous variables were expressed as median ± standard deviation or interquartile range and categorical variables were expressed as numbers or percentages. (b) Student's *t*-test, unpaired Mann–Whitney *U* test, or chi-squared test was performed where appropriate. NAFLD: nonalcoholic fatty liver disease, BMI: body mass index, VFA: visceral fat area, WC: waist circumference, FPG: fasting plasma glucose, FINS: fasting insulin, TG: triglyceride, HDL-C: high-density lipoprotein cholesterol, LDL-C: low density lipoprotein cholesterol, ALT: alanine aminotransferase, AST: aspartate aminotransferase, UA: uric acid, TyG: triglyceride and glucose, LAP: lipid accumulation product, VAI: visceral adiposity index, and THR: triglyceride to high-density lipoprotein cholesterol ratio.

**Table 2:** Univariate logistic regression analysis of factors affecting IR.

Characteristics	OR	95% CI	Wald *χ*^2^	*P*
Female	1			
Male	0.899	0.628–1.259	0.439	0.507
Age (y)	0.998	0.982–1.014	0.076	0.782
Smoking, *n* (%)	0.768	0.554–1.084	2.259	0.133
Drinking, *n* (%)	0.860	0.597–1.240	0.650	0.420
Hyperuricemia, *n* (%)	1.639	0.831–3.232	2.033	0.154
Dyslipidemia, *n* (%)	1.386	0.982–1.957	3.441	0.064
NAFLD, *n* (%)	1.964	1.379–2.796	14.011	<0.001
Hypertension, *n* (%)	0.916	0.587–1.429	0.150	0.698
BMI (kg/m^2^)	1.177	1.116–1.241	36.311	<0.001
VFA (cm^2^)	1.018	1.012–1.025	31.913	<0.001
WC (cm)	1.052	1.034–1.070	32.910	<0.001
FPG (mmol/L)	1.312	1.210–1.422	43.424	<0.001
FINS (*μ*U/mL)	1.907	1.701–2.138	122.162	<0.001
TG (mmol/L)	1.365	1.156–1.613	13.456	<0.001
HDL-C (mmol/L)	1.166	0.686–1.981	0.322	0.570
LDL-C (mmol/L)	1.177	0.951–1.456	2.249	0.134
ALT (IU/L)	1.019	1.009–1.030	12.751	<0.001
AST (IU/L)	1.026	1.007–1.046	7.438	0.006
UA (*μ*mol/L)	1.003	1.000–1.005	5.447	0.020

IR: insulin resistance; OR, odds ratio, CI: confidence interval, NAFLD: nonalcoholic fatty liver disease, BMI: body mass index, VFA: visceral fat area, WC: waist circumference, FPG: fasting plasma glucose, FINS: fasting insulin, TG: triglyceride, HDL-C: high-density lipoprotein cholesterol, LDL-C: low density lipoprotein cholesterol, ALT: alanine aminotransferase, AST: aspartate aminotransferase, and UA: uric acid.

**Table 3:** Multivariate logistic regression analysis of factors affecting IR.

Characteristics	OR	95% CI	Wald *χ*^2^	*P*
Age (y)	1.028	1.008–1.048	7.380	0.007
VFA (cm^2^)	1.016	1.009–1.023	18.893	<0.001
TG (mmol/L)	1.184	1.005–1.396	4.062	0.044
FPG (mmol/L)	1.334	1.225–1.451	44.466	<0.001
ALT (IU/L)	1.021	1.001–1.040	4.334	0.037
AST (IU/L)	0.994	0.961–1.029	0.115	0.735
UA (*μ*mol/L)	1.002	0.999–1.004	1.888	0.169
NAFLD, *n* (%)	1.273	0.840–1.929	1.296	0.255

IR: insulin resistance, OR: odds ratio, CI: confidence interval, VFA: visceral fat area, FPG: fasting plasma glucose, TG: triglyceride, ALT: alanine aminotransferase, AST: aspartate aminotransferase, UA: uric acid, and NAFLD: nonalcoholic fatty liver disease.

**Table 4:** The AUC, sensitivity, specificity, and cut-off points for each index for predicting IR.

Indexes	AUC		Sensitivity		Specificity		Cut-off values	
	Derivation cohort	Validation cohort	Derivation cohort	Validation cohort	Derivation cohort	Validation cohort	Derivation cohort	Validation cohort
Predictive model	0.77	0.91	0.62	0.82	0.81	0.89	8.18	7.46
TyG index	0.69	0.84	0.81	0.86	0.48	0.77	1.60	0.81
LAP	0.66	0.89	0.81	0.86	0.46	0.82	33.54	42.48
VAI	0.63	0.85	0.86	0.86	0.40	0.72	1.44	1.20
THR	0.53	0.83	0.70	0.84	0.41	0.75	1.14	0.73

IR: insulin resistance, AUC: area under the curve, TyG: triglyceride and glucose, LAP: lipid accumulation product, VAI: visceral adiposity index, and THR: triglyceride to high-density lipoprotein cholesterol ratio.

## Data Availability

The data analyzed during the current study are available from the corresponding author upon reasonable request.

## Supplementary Materials

Supplementary Materials STROBE checklist for this cross-sectional study.
